# The efficacy of Ksharsutra, Fistulectomy and Ligation of Intersphincteric Fistula Tract (LIFT) procedure in management of Fistula in ano a prospective observational study

**DOI:** 10.1186/s12893-023-01969-w

**Published:** 2023-03-29

**Authors:** Chinniahnapalaya Pandurangaiah Hariprasad, Anil Kumar, Manoj Kumar, Manoj Kumar, Shiv Shankar Paswan, Gupta Rohit, Shiv Kishor, Prem Kumar

**Affiliations:** 1 Department of General Surgery, All India Institute of Medical Sciences, Patna, India; 2 Department of Radiodiagnosis, All India Institute of Medical Sciences, Patna, India

**Keywords:** Ksharsutra, Fistulectomy, Ligation of intersphincteric fistula tract, Fistula in Ano

## Abstract

**Background:**

Fistula in ano is always a troublesome condition for the clinician and the patients owing to its complexity, recurrences, and high morbidity since ancient times. There is no gold standard treatment modality to date documented in the literature for complex fistula in ano.

**Material and methods:**

We enrolled 60 consecutive adult patients attending the surgical outpatient department of a tertiary care centre in India, diagnosed with complex fistula in ano. Among them, 20 each in the Ligation of intersphincteric fistula tract (LIFT), Fistulectomy andKsharsutra(Special medicated seton) group were respectively recruited randomly. A prospective observational study was conducted. The primary outcomes were postoperative recurrence and morbidity. Post-operative morbidity is measured in terms of postoperative pain, postoperative bleeding, pus discharge and post-operative incontinence. The result of the study were analysed after 6 months of follow-up by clinical examination at outpatient department and at 18 months follow up done telephonically.

**Results:**

At 6 months of follow-up, 2 patients (10%) had a recurrence in the Ligation of intersphincteric fistula tract procedure group, 3 patients (15%) in the fistulectomy group and 6 patients (30%) in Ksharsutra group, however 3(15%), 4(20%) and 9(45%) patients developed recurrence in Ligation of Intersphincteric fistula tract, Fistulectomy and Ksharsutra group respectively at 18 month of follow-up. The differences in the recurrence were not statistically significant.The mean Visual analogue score for postoperative pain after 24 h as well as after 48 h were statistically significant in Ligation of intersphincteric fistula tract versus Ksharsutra group (*p* < 0.05). The Visual analogue score for post–operative pain was also significant in the Ligation of the intersphincteric fistula tract versus the Fistulectomy group (*p* < 0.05). The patients treated via Fistulectomy and Ksharsutra had a higher proportion of bleeding (15%) as compared to the Ligation of intersphincteric fistula tract procedure. Postoperative morbidity was statistically significant between the Ligation of intersphincteric fistula tract versus the Ksharsutra and the Ligation of intersphincteric fistula tract versus Fistulectomy.

**Conclusion:**

Ligation of intersphincteric fistula tract had less postoperative morbidity compared to Fistulectomy and Ksharsutra procedure; although recurrence was less compared to other methods it was statistically not significant.

## Introduction

Fistula in ano is defined as abnormal tract lined by the granulation tissue which runs between the external openings in the perineum to an internal opening located in the anorectal canal [1]. Prevalence of Fistula in ano is varied it is 1.2 to 2.8/10000 population in European countries [2]. This disease comes under Ashtamahagdas under eight grave disorders described by Sushruta in ancient documentation of surgery in India [3]. Ligation of Intersphincteric Fistula Tract (LIFT) was originally described in 2007 and is based on principle of ligating in the fistulous tract in the Intersphincteric plane and closing the internal opening [4]. Ksharsutra is a modality of treatment of fistula in ano since ancient times in India. Ksharsutra is a type of Seton placed after cannulating and probing of the fistulous tract without surgically excising the tract. Ksharsutra induces sclerosis in the fistulous tract after the tract has been drained [5]. It is medicated thread prepared by smearing Kshar of Apamarga (Achyranthus aspera), Haridra (curcuma Longa) and Shnuhi (Eforbia Nerrifolia) [6]. The Ksharsutra works as draining and cutting seton with simultaneously promoting the process of healing. Ksharsutra is inserted using a metallic probe passing through the external opening and thread tied to the tail end of the probe and probe advanced through the tract to exit out through the external opening. At regular interval of 2 weeks, the ksharsutra requires to be changed and progressively tightened it helps in ischemic necrosis of the muscle complex and promoting fibrosis of the fistula tract [7]. Fistulectomy is a procedure where the entire probed tract will be tried be excised. The treatment procedure of perianal fistula aims at complete eradication of sepsis while preserving the continence. Fistulotomy which involves the lay open of fistula tract has shown promising results for simple fistula in ano [8]. Treatment of Complex fistula in ano is still a complex task even with advent of newer techniques like expanded adipose-derived stem-cells (ASCs), Video-assisted anal fistula treatment (VAAFT) especially in resource limited setting [9]. Procedures like LIFT, Fistulectomy and application of Ksharsutra being technically less demanding, identification of better treatment procedure would benefit in treatment of complex fistula in ano in terms of reduced recurrence and reduced postoperative morbidity in source limited settings.

## Material and methods

**Fig. 1:**
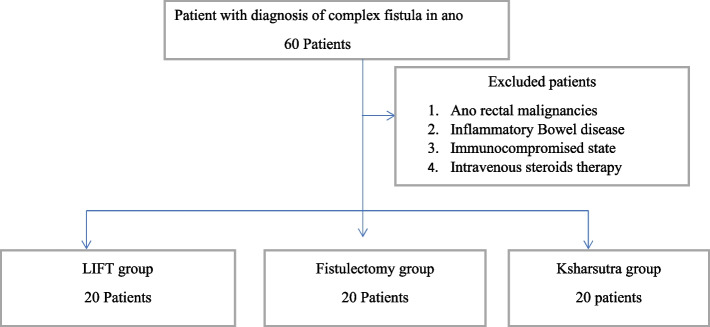
Algorithm that shows patient selection

We analysed the data of 60 consecutive patients’ diagnosed of complex fistula, which underwent LIFT, Ksharsutra placement and Fistulectomy during the study period between December 2019 to June 2021. Complex fistula-in-ano was defined as fistula with the tract crossing more than 30% of the sphincter complex, recurrent or with multiple tracts. The objective of the study was to assess the post-operative recurrence and morbidity of LIFT, Ksharsutra and Fistulectomy. The Patients with clinical and investigative evidence of ano-rectal malignancy, fistulas due to Inflammatory Bowel Disease, active Tuberculosis, immunocompromised, previous radiation therapy, patients on Intravenous Steroids, uncontrolled Diabetes mellitus were excluded from the study. Patient selection depicted in Fig. 1.

All patients underwent comprehensive clinical examination (digital rectal examination and proctoscopy) and Magnetic Resonance Imaging Fistulogram (MRI Fistulogram). Patients were randomly divided into three groups with 20 patients each in series of outpatient vist, however each group were homogenised with respect to age, gender, comorbidities, previous surgical interventions, grade of fistula in Ano.Post operatively pain was assessed on visual analogue scale (average from 0–10) after 24 h and 48 h of surgery and on 7th post-operative day with fixed dose of analgesic in each group. Post-operative pus discharge, and bleeding were assessed on first post-operative day, 7th post-operative day and then on monthly basis for 6 months. Incontinence for flatus and stool was assessed by Browning and Park’s incontinence scale [10] on first post-operative day, 7th post-operative day and then on monthly basis for 6 months. Recovery was defined as closure of wounds, fistulous orifices, and absence of suppuration. Recurrence was diagnosed as failure of complete wound healing with persistent external opening, and new external opening.. The follow up at 18 months was done telephonically and the patient were asked regarding any complaint of new onset of perianal discharge, pain or incontinence to flatus, stool or both. The number of times and timing of ksharsutra tightening in case of ksharsutra placement was noted. Recovery and recurrence in case of Ksharsutra procedure was noted after removal of Ksharsutra.

### Statistical analysis

Sample size was calculated using powerandsamplesize.com, using the reference from the previous study [11]. Descriptive statistics was used for qualitative data. The chi square test and one-way Anova test was used for comparison of categorical data. The data analysis was done using IBM-SPSS version 22.

## Results

In our study majority of the patients were men 51 (85%) and only 9 patients were women (15%). The duration of symptom was noted to be mean ± SD (13.33 SD 8.402 months). The minimum duration of symptoms was 2 months and maximum duration 36 months. The patients demographic data has been depicted in (Table 1).

**Table 1:** Distribution of patients according to patient characteristics (*N* = 60)

Variables	N	Percentage (%)
Gender		
Men	51	85
Women	9	15
Comorbidities		
Hypertension	6	10.0
Type 2 Diabetes	6	10.0
Mellitus		
Tuberculosis	1	1.7
No comorbidities	47	73.8
Addictive Habits		
Tobacco chewing	17	28.3
Smoking	17	28.3
Alcohol Consumption	03	5.0
No Addictive habits	23	38.3

Previous surgical intervention like draining of perianal abscess by incision and drainage was done in 6 (10.0%) patients. More than half of patients had external opening posteriorly 46(76.7%) followed by anteriorly 11(18.3%) and laterally 3(5%).. The mean length of the fistula tract was 3.68SD1.071 cm with minimum length of 2 cm and maximum length of 6 cm. (Table 2).

**Table 2:** Distribution of patients according to patient according to clinical examination parameters of fistula in ano

Variables	*N* = 60	Percentage (%)
Previous Surgical interventions		
Incision and drainage	6	10.0
No Previous Surgery	54	90.0
Position of External opening		
Posterior	46	76.7
Anterior	11	18.3
Lateral	3	5.0
Parks classification of Fistula		
Intersphincteric	28	46.7
Trans-sphincteric	32	53.3

A chi-square test for association was conducted between the procedure done and recurrence at 2, 3 and 6 months and 18 months. There were 7 out of 9 expected cell frequencies lesser than five. Hence, Fischer’s exact t-test was applied. The patients treated with Ksharsutra had a higher proportion of recurrence as compared to the other two procedures. There was no statistically significant association between the surgical procedure done and recurrence in all three groups, there was a moderately strong association between recurrence and the procedure done at 2 and 3 months and a weak association between both the variables at 6 months and 18 months, but these associations were found to be statistically insignificant in all three groups, as shown in Table 3.

**Table 3:** Recurrence at the end of 2, 3, 6 and 18 months( Telephonically) of follow up

Recurrence	LIFT (*n* = 20)	Fistulectomy (*n* = 20)	Ksharsutra (*n* = 20)	*N* = 60
Recurrence at 2 months	1 (5%)	2 (10%)	3 (15%)	6 (10%)
Recurrence at 3 months	2 (10%)	3 (15%)	5 (25%)	10(16.66%)
Recurrence at 6 months	2 (10%)	3 (15%)	6 (30%)	11 (18.33%)
Recurrence at 18 months	3 (15%)	4 (20%)	9 (45%)	16(26.6%)

At 2 months—Cramer’s V = 0.136, *p* = 0.574

At 3 months – Cramer’s V = 0.167, *p* = 0.432

At 6 months – Cramer’s V = 0.220, *p* = 0.235

At 18 months – Cramer’s V = 0.297, *p* = 0.071

A one-way ANOVA was conducted to determine if the post-operative pain Visual Analogue Score (VAS) scores were different for groups with different procedures. There was an increase in the VAS scores in the three groups – LIFT, Fistulectomy and Ksharsutra procedure in that order as shown in Table 4.

**Table 4:** Comparison of VAS scores among three procedures – LIFT, Fistulectomy and Ksharsutra

	LIFT	Fistulectomy	Ksharsutra	F(df)	*p*-value
	Mean ± SD				
VAS^*^ scores after 24 h	2.8 ± 1	3.6 ± 1.18	3.75 ± 1.20	4.032	0.023
VAS scores after 48 h	2.05 ± 0.82	3.15 ± 1.04	3.30 ± 1.03	9.889	0.001
VAS scores after 7 days	1.5 ± 0.60	2.45 ± 0.94	2.5 ± 0.68	10.985	0.001

^*^VAS Visual analogue score

A chi-square test for association was conducted between the procedure done and bleeding at Post-operative day (POD) 1 and POD 7. All 6 expected cell frequencies were lesser than five. Hence, Fischer’s exact value was taken. The patients treated via Fistulectomy and Ksharsutra had a higher proportion of bleeding (15% each) as compared to the LIFT procedure. The Fischer’s exact value (F value = 6.029) was significant in the POD 7 group; (*p* = 0.03).as shown in (Table 5).

**Table 5:** Comparison of bleeding at POD 1 and POD 7

Bleeding ( +)	Procedure performed			Fischer exact value	*p*-value
	**LIFT**	**Fistulectomy**	**Ksharsutra**		
POD^*^ 1	1 (5%)	3 (15%)	3 (15%)	1.374	0.68
POD 7	0	0	4 (20%)	6.029	0.03

^*^POD Post- operative day

A chi-square test for association was conducted between the procedure done and incontinence for flatus and stool at 1 month, 2 months, 3 months, 4 months, 5 months and 6 months and 18 months The patients treated with Ksharsutra procedure had the highest proportion (30%) of patients presenting with incontinence as compared to the other two procedures – Fistulectomy (15%) and LIFT (5%). But this was not statistically significant (*p* > 0.05), as shown in Table 6.

**Table 6:** Comparison of incontinence for flatus and stool at various follow up periods; (*N* = 60)

**Incontinence grade (grade 2)**	**Procedure performed**				**Fischer exact value**
	**LIFT (*n* = 20)**	**Fistulectomy (*n* = 20)**	**Ksharsutra (*n* = 20)**	*N* = 60	
1 month	0	1 (5%)	1 (5%)	2 (3.33%)	1.27
2 months	0	2 (10%)	2 (10%)	4 (6.66%)	2.19
3 months	0	2 (10%)	3 (15%)	5 (8.33%)	3.03
4 months	0	2 (10%)	2 (10%)	4 (6.66%)	2.19
5 months	0	2 (10%)	3 (15%)	5 (8.33%)	3.03
6 months	0	2 (10%)	4 (20%)	6 (10%)	4.23
18 months	1 (5%)	3 (15%)	6 (30%)	10 (16.6%)	4.27

## Discussion

Extensive research is being conducted to devise a gold standard technique for treating Fistula in ano. In a metanalysis of the randomized control trials which have compared fistulotomy and fistulectomy involving six randomized trials with 565 patients, they did not find any statistically significant difference between the two groups in terms of postoperative complications and recurrence rate and incontinence [12].

### Clinical profile

The mean duration of symptoms in our study was 13.33 SD 8.4 months and more than half of the patients (53.3%) presented to us with the clinical symptom of pain and perianal discharge. In our study 6 (10%) have previously undergone incision and drainage for the perianal abscess. Majority of our patients had external opening located posteriorly 46 (76.7%). We have found a similar observation in a previously conducted study that majority 65% of the patients had location of external opening of fistulous tract in the posterolateral [13]. In our study all our patients had single external opening of fistulous tract. Majority of our patients 60% had excoriation of the surrounding skin on examination of the perianal examination. The mean length of fistula tract was 3.68 SD 1.07 cm.

### Postoperative Morbidity

- **a) Postoperative pain**
  In our study pain score was recorded using visual analogue score at regular intervals after 24 h, 48 h and after 7 days. The mean overall VAS score at 24 h was noted to be 3.38 SD 1.19, at 48 h 2.83 SD 1.10 and after 7 days it was recorded to be 2.15 SD 0.88. The mean VAS scores after 24 h were statistically significant in LIFT (2.8 SD 1) versus Ksharsutra group (3.75 SD 1.2) (*p* < 0.05). VAS scores after 48 h were statistically significant in the LIFT (2.05 SD 0.82) versus Ksharsutra group (3.30 SD 1.03) (*p* < 0.05) and in the LIFT (2.05 SD 0.82) vs Fistulectomy group (3.15 SD 1.04) (*p* < 0.05). Previous study in contradictory to our study noted that the mean pain VAS score after 24 h among Fistulectomy (7.89 SD 0.76) versus Ksharsutra (5.38 SD 0.69) (*p* < 0.05) was statistically significant also after 48 h mean VAS score between Fistulectomy (4.18 SD 0.42) versus Ksharsutra (2.48 SD 0.5) (*p* < 0.05) [13]. On comparison of VAS scores after 7 days, again, the mean scores were statistically significant in the LIFT (1.5 SD 0.6) versus Ksharsutra groups (2.5 SD 0.68) (*p* < 0.05) and in the LIFT (1.05 SD 0.6) vs Fistulectomy groups (2.45 SD 0.94) (*p* < 0.05). In the previous study the mean pain score on postoperative day 1 between LIFT (6.72 ± 0.53) versus Fistulectomy (7.01 ± 0.56) (*p* < 0.05), on postoperative day 3 LIFT (5.66 ± 0.11) versus Fistulectomy (6.01 ± 0.11) (*p* < 0.05) and on postoperative day 5 LIFT (2.43 ± 0.24) versus Fistulectomy (3.51 ± 0.25) (*p* < 0.05). The results of this study were in concordant to our study [14].
- b) Postoperative pus discharge and bleeding
  Postoperative pus discharge and bleeding was noted at various intervals postoperatively in our study. The patients treated with Fistulectomy and Ksharsutra had a higher proportion of bleeding (15% each) as compared to the LIFT procedure. The Fischer’s exact value (F value = 6.029) was significant in the POD 7 group; (*p* = 0.03). This observation might be due to the more invasiveness of these procedures like fistulectomy involves the removal of the entire fistulous tract leading to more tissue damage in the perianal region which may contribute to the bleeding from the surgical site. Ksharsutra on other hand is Seton which cuts through the muscles and perianal tissue leading to increased chance of postoperative bleeding. In one of the previous studies noted postoperative bleeding among 5.4% of individuals who were undergoing fistulectomy and none among Ksharsutra and LIFT category [11]. Postoperative pus discharge was noted among 16.7% of the patients undergoing LIFT and none among fistulectomy group. This result was consistent with our study results. In a study in comparing Fistulectomy and fistulotomy with marsupialization and noted that persistent wound discharge was documented up to period of 4 weeks in the fistulectomy group and up to about 2 weeks in group of Fistulectomy with marsupialization [15]. A study to compare Fistulectomy versus Fistulectomy reported bleeding from the surgical site in about 12% of the patients in the postoperative period which is similar to our finding [16].
- c) Postoperative incontinence
  Postoperative incontinence in our study was noted at various intervals using Browning and Park’s incontinence scale. The patient treated with Ksharsutra procedure (30%) had highest proportion of grade 2 (incontinence to flatus) compared to other two groups Fistulectomy (15%) and none in the LIFT group but the difference was not statistically significant (*p* > 0.05). None of the patients had higher grades of incontinence (incontinence to liquid and stools). In a comparative study of fistulectomy versus LIFT noted incontinence of about 16.2% in fistulectomy group and 2.1% in the LIFT group in this study results however simulated our study results the scale used to record incontinence was Wexner incontinence score (WIS) unlike in our study Browning and parks scale was used [17]. In a study on Ksharsutra procedure in treatment of fistula in ano noted minor incontinence of 14%, however in this study no definitive standard scale was used in the documentation of the incontinence [18]. In a study to compare fistulotomy and fistulectomy for simple fistula in ano noted postoperative minor incontinence among 5 out of total 44 subjects which accounted to 11.36%, although the minor incontinence was defined as incontinence to flatus and occasional incontinence to liquids clear scaling system was not incorporated [19]. In a study conducted evaluate the effectiveness of LIFT procedure noted no incontinence in the immediate postoperative period but after 3 years of follow up they noted incontinence to flatus among 8 out of 25 patients, incontinence to liquids among 4 out of 25 patients and incontinence to solid in 7 out of 25 patients however our study did not show any incontinence in LIFT group [20].

### Postoperative recurrence

Recurrence was the primary outcome measure in our study and we have defined recurrence as complete wound healing failed with persistent external opening after 8 weeks of surgery or new external opening was found during follow up. Postoperative recurrence was noted in all follow up visit in our study, at the end of 18 months of follow up, 3 patients (15%) had recurrence in LIFT procedure group and 4 patients (20%) in the fistulectomy group and 9 (45%) among Ksharsutra group, however the difference between the recurrence between the groups were not statistically significant. In a study comparing Ksharsutra and Fistulectomy did not note any recurrence in both the groups at 6 months of follow up; however at 1 year of follow up recurrence rate among fistulectomy group was 7.14% as compared to ksharsutra group (2.38%) which was statistically significant [13]. This study showed ksharsutra had low recurrence rate as compared to fistulectomy group which is not in concordance to our study. In a comparative study of LIFT versus Fistulectomy noted recurrence of 12% of recurrence in the LIFT group at the end of 10 month follow up [21].In a study to evaluate the efficacy of Ksharsutra noted recurrence rate of 3.33% at a follow up period of 4 years [22]. Larger meta-analysis and systematic reviews have showed LIFT procedure reduced morbidity and recurrence [23, 24]. Our study also favours LIFT procedure owing to its nature of sphincter saving procedure with minimal tissue injury. Setons like Ksharsutra procedure have classically shown to have greater morbidity and prolonged healing time.

### Limitations

- 1. Smaller sample size limits us in generalizing the results
- 2. We appreciate that the follow up period should have been more for better interpretation of results.
- 3. We acknowledge that a larger study group which has been randomized in to three groups would have yielded robust results.

## Conclusion

This study was conducted to compare the efficacy of Ksharsutra, Fistulectomy and Ligation of Intersphincteric Fistula Tract (LIFT) procedures in management of fistula in ano. Although recurrence rate was less in the LIFT group as compared to Ksharsutra and Fistulectomy groups, the difference was not statistically significant. Post operative morbidity assessed in terms post operative pain, postoperative pus discharge, postoperative bleeding and postoperative incontinence to flatus and stool among which Post operative was statistically significant between LIFT versus Ksharsutra and LIFT versus Fistulectomy.

## Data Availability

The data that support the findings of this study are available from the corresponding author, Dr Anil Kumar, upon reasonable request.
